# Estimated Transmissibility and Case Fatality Rate of Bundibugyo Virus, Uganda, 2007

**DOI:** 10.3201/eid3210.261175

**Published:** 2026-10

**Authors:** Bianca de Padua, Andrei R. Akhmetzhanov

**Affiliations:** National Taiwan University College of Public Health, Taipei, Taiwan

**Keywords:** viruses, Bundibugyo virus, reproduction number, case-fatality rate, modeling, Uganda

## Abstract

Because the epidemiology of Bundibugyo virus remains unclear, we reanalyzed the first recognized outbreak (Uganda, 2007). Adjusting for case under-ascertainment and the effect of control measures, we estimated the effective reproduction number (1.55, falling to <1 after intervention) and case-fatality rate (31%, declining to 25%). The underascertainment rate was 15%.

Bundibugyo virus (BDBV) is 1 of 6 orthoebolaviruses (genus *Orthoebolavirus*), constituting the species *Orthoebolavirus bundibugyoense* (*1*) and causes rare but severe disease in humans (*2*). Two other orthoebolaviruses, Ebola virus (EBOV) and Sudan virus, have caused the largest outbreaks and are highly lethal; case-fatality rates (CFRs) average ≈50% (*2*). BDBV is considered less lethal; CFRs are reported to be 30%–40%, and it has historically caused only small outbreaks (*3*). The ongoing 2026 outbreak in the Democratic Republic of the Congo and Uganda shows a different pattern: first reports in late May 2026 recorded >600 suspected cases, and >2,000 cases were projected within 3 months (*4*). Despite this potential for large outbreaks, the transmissibility and severity of BDBV remain poorly characterized.

Control measures, such as case isolation, active case finding, infection control in hospitals and isolation wards, and safe burial practices, are key to curbing Ebola outbreaks, in part by limiting superspreading and lowering mortality rates (*5*). However, those measures also alter the epidemiologic parameters used to characterize an outbreak: the desirable reduction in the reproduction number can be accompanied by improved case ascertainment and reduced mortality rates. Failing to account for the effect of control measures can bias estimates of transmissibility and severity, whereas adjusting for them yields values closer to the natural history of the virus. In this study, we reanalyzed the first reported BDBV outbreak (Bundibugyo, Uganda, 2007) (*6*) to obtain robust estimates of transmission potential and CFR adjusted for both case underascertainment and the effect of control measures.

We digitized weekly counts of confirmed and probable cases from a 2010 study (*6*), retaining 110 cases (37 deaths) with a known week of symptom onset. We excluded 6 additional cases (2 deaths) lacking onset dates. Those data are consistent with 116 confirmed and probable cases and 39 deaths (crude CFR 34%) reported for the outbreak (*6*). Control measures were initiated in week 48, which we used to define preintervention and postintervention periods. We estimated transmission with a renewal process incorporating intrinsic back-projection to the time of infection (*7*), informed by the incubation period and time from onset to transmission, the latter adapted from EBOV (*8*). That linked the effective reproduction number (R_t_) to the time of infection rather than to symptom onset. We modeled the intervention as a step reduction at week 48 in R_t_ and in case underascertainment, together with a multiplicative reduction in the CFR. We assumed that deaths were fully recorded throughout the outbreak, whereas nonfatal cases that occurred before the intervention could have been missed (*9*). We fitted the model in a Bayesian framework and report posterior means with 95% credible intervals (CrIs) (Appendix). 

Before the intervention, BDBV was capable of sustained transmission of R_t_ of 1.55 (95% CrI 1.02–2.36). The R_t_ markedly declined to 0.50 (95% CrI 0.17–1.15) after the intervention (Figure; Appendix Figure 1). We estimated that ≈15% (95% CrI 2.7%–31.7%) of cases were under ascertained, corresponding to 20 (95% CrI 3–51) cases predicted to be missed. After adjusting for underascertainment, the preintervention CFR was 31.4% (95% CrI 21.8%–42.8%), which then decreased to 25.1% (95% CrI 16.3%–34.6%), which translated to an odds ratio of death (postintervention vs. preintervention) of 0.75 (95% CrI 0.40–0.99). Those estimates were robust to the time variation in case-ascertainment during the preintervention period, and posteriors deviated substantially from the priors (Appendix Figures 2–5).

**Figure:**
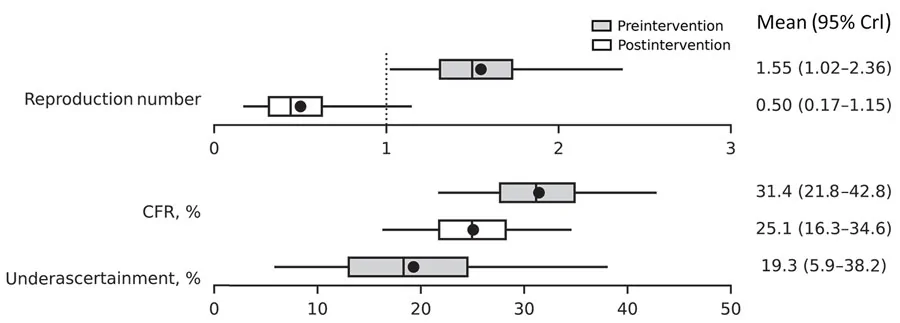
Estimates of epidemiologic parameters from study of transmissibility and case fatality rate of Bundibugyo virus estimated from first outbreak, Uganda, 2007. Estimates are stratified by preintervention and postintervention periods. Each boxplot displays the interquartile range (box left and right edges), 95% CrI (whiskers), median (vertical line), and mean (solid circle). Dotted vertical line indicates the threshold reproduction number of 1. CFR, case-fatality rate; CrI, credible interval.

Our findings suggest that BDBV is moderately transmissible; its preintervention reproduction number was ≈1.5 and its CFR ≈31%. That finding places BDBV within the range of other orthoebolaviruses, although with a lower CFR than typical EBOV outbreaks. The R_t_ fell to <1 after the outbreak response, and the fatality risk also declined (the 95% CrI of the odds ratio excluded 1). A reduction in transmission of ≈35% sufficed to bring the R_t_ to <1 and was achieved by nonpharmaceutical interventions alone in 2007. Such control measures remain operationally demanding, as the 2026 outbreak shows. A serosurvey among close contacts of survivors conducted 29 months after the 2007 outbreak identified 13 symptomatic infections that had not been recorded (*10*), implying that 21.0% of symptomatic infections were unascertained. That lies within the CrI of our estimate of ≈15% of missed cases, though above our point estimate. 

The primary limitation of our study is that it was based on a single small outbreak and used counts digitized from a previously published article, and no BDBV-specific epidemiologic intervals were available. Estimates depended on the assumed time from onset to transmission, the transmission-model structure, and assumptions about death ascertainment. Some CrIs were wide, particularly for the postintervention R_t_ and the under-ascertainment rate, reflecting the small number of postintervention weeks and the limited information on unascertained cases. Our model also did not reproduce the peak in week 48, which could reflect superspreading (Appendix). Given the scarcity of epidemiologic data on BDBV, our estimates provide a basis for future modeling. Nonetheless, more accurate BDBV-specific estimates of the epidemiologic time intervals and transmission parameters are still needed.

Appendix Additional information about transmissibility and case fatality rate of Bundibugyo virus estimated from first outbreak, Uganda, 2007.
