# Supplementary material for: Estimated Transmissibility and Case Fatality Rate of Bundibugyo Virus, Uganda, 2007
Source: Emerg Infect Dis. 2026 Oct;32(10):1762–4. doi: 10.3201/eid3210.261175 (PMC13632865; doi:10.3201/eid3210.261175)
Supplement: Supplementary file 1 [file 26-1175-Techapp-s1.pdf]

Article DOI: https://doi.org/10.3201/eid3210.261175

EID cannot ensure accessibility for supplementary materials supplied by authors. Readers who have difficulty accessing supplementary content should contact the authors for assistance.

Estimated Transmissibility and Case Fatality Rate of Bundibugyo Virus, Uganda, 2007

Appendix

Table of Contents

1. Data	**1**
2. Modeling framework	2
2.1. Epidemiologic time intervals	2
2.2. Discretization scheme	3
2.3. Transmission dynamics	5
2.3.1. Renewal process with intrinsic backprojection	5
2.3.2. Adjusting for the effect of control measures	6
2.4. Model specifications	7
2.4.1. Semi-mechanistic model	8
2.4.2. Fully mechanistic model	9
2.4.3. Gamma approximation of the binomial for estimating CFR	10
2.4.4. Partial ascertainment of both cases and deaths	11
2.4.5. Choice of priors	12
3. Sensitivity analysis	12
3.1. Model variations	12
3.2. Time-varied case under-ascertainment	14
3.3. Partial ascertainment of both cases and deaths	15
4. Technical details	15
5. Model diagnostics	15
6. AI disclosure	16
7. Study limitations	16
**R**eferences	17
Appendix tables	20
Appendix figure captions	23

1. Data

Individual-level line-list data for the 2007 Bundibugyo virus (BDBV) outbreak were not publicly available; we therefore reconstructed the weekly incidence by digitizing Figure 2 of Wamala et al. (*1*). The figure presents probable and confirmed cases by week of symptom onset,

stratified by outcome; for each epidemiologic week we recorded the number of fatal and non-fatal cases.

The extracted series comprised 110 cases (37 fatal, 73 non-fatal) with onset during epidemiologic weeks 30–52 of 2007. This total is slightly lower than the 116 probable and confirmed cases reported in the text of Wamala et al.; the 6-case difference (including 2 deaths) corresponds to cases not represented in the epidemic curve. The case-fatality rate of the extracted series (34%) matched that of the full case series. The outbreak was laboratory-confirmed and strict isolation measures were initiated during epidemiologic week 48, which we used to define the pre- and postintervention periods. To address potential overestimation of the postintervention effective reproduction number, we added 3 weeks of trailing zeros after week 52, so that the subsequent absence of cases contributed to the likelihood.

2. Modeling framework

2.1. Epidemiologic time intervals

Owing to the considerable uncertainty about the natural history of Bundibugyo virus disease (BVD) at the time of this study, we relied on the limited available information and, when no information could be found, on previous estimates for Ebola virus disease (EVD) (*2*), , a disease closely related to BVD. Our model required two input distributions: the incubation period and the time from symptom onset to transmission (TOST), the latter also known as the profile of infectiousness.

We modeled the incubation period, with probability density f, using a gamma distribution. We used the documented central characteristics and ranges to determine the parameters of the gamma distribution. As Wamala et al. (*1*) reported a median and a range, another source (*3*) noted an average range of 4–10 days. We fitted these quantities with the range interpreted as the 95% confidence interval and the average range as the interquartile range (IQR) to a gamma distribution using the R package *rriskDistributions* (version 2.1.2; function get.gamma.par). The resulting mean was estimated at 7.71 days and standard deviation (SD) 4.68 days (shape 2.72, rate 0.35 day^{-1}; median 6.79 days), consistent with the incubation period reported for EVD outbreaks (*2*).

For the sensitivity analysis, we also used Weibull and lognormal distributions fitted to the same ranges (Weibull: mean 7.48 days, SD 4.18 days, shape 1.86, scale 8.42 via get.weibull.par function; lognormal: mean 8.19 days, SD 6.01 days, meanlog 1.89, sdlog 0.66 via get.lnorm.par function). We also tested the role of the shape of the distribution on the estimates by using a triangular distribution, which required only minimal assumptions about the shape. Wamala et al. (*1*) reported a median incubation period of 7 days (range 2–20 days), which we represented as a triangular distribution with a mode of 7 days, a minimum of 2 days, and a maximum of 20 days. The resulting mean of 9.7 days is close to the mean incubation period reported for EVD outbreaks (*2*). Finally, we also considered the known estimates of the incubation period for EVD modeled by a gamma distribution and fitting the data for the 2013–2016 West Africa EVD outbreak, when the mean was estimated at 11.4 days and SD was estimated at 5.2 days (*4*).

We adopted the TOST distribution, with density λ, from the literature on EVD. Yamin et al. (*5*) estimated the risk of infection as a function of time since symptom onset, which defines the TOST distribution. We extracted the data points from Figure 1 (bottom panel) of Yamin et al. and fitted gamma distributions to survivors and nonsurvivors. The estimated mean TOST was 4.8 days for survivors and 4.9 days for nonsurvivors, with SDs of 2.4 days and 2.2 days, respectively. The two means did not differ considerably, consistent with the findings of Yamin et al. that the risk of infection does not differ between survivors and nonsurvivors. Hence, we used the gamma distribution fitted to survivors in our model. We also investigated the effect of varying the mean of the TOST distribution on the model estimates by considering three means at 3, 4.5, and 6 days, while keeping the SD the same.

2.2. Discretization scheme

We modeled the transmission dynamics at a daily scale which required discretizing both the incubation period and the TOST into daily intervals, using the centered discretization scheme (*6*). Let t denote the number of days since the reference event (exposure for the incubation period, or symptom onset for the TOST) and let F and Λ be the corresponding cumulative distribution functions (in days). For the incubation period, we treated onset on the day of exposure ($t = 0$), or beyond $t_{\max} = 28$ days as negligible:

$$f_t = \begin{cases} \dfrac{F(t+0.5) - F(t-0.5)}{F(t_{\max}+0.5) - F(0.5)}, & t = 1, \dots, t_{\max} \\ 0, & \text{otherwise} \end{cases}$$

The TOST distribution was discretized analogously, while retaining transmission on the day of symptom onset ($t = 0$) and truncating beyond $t_{\max}^{\text{ost}} = 21$ days (3 weeks)

$$\lambda_t = \begin{cases} \dfrac{\Lambda(t + 0.5) - \Lambda(t - 0.5)}{\Lambda(t_{\max}^{\text{ost}} + 0.5)}, & t = 0,1,2,\dots,t_{max}^{ost} \\ 0, & \text{otherwise} \end{cases}$$

Because transmission is assumed to be post-symptomatic, $\Lambda(-0.5) = 0$ and the lower term of the normalizing denominator vanishes. For the sensitivity analysis we also investigated the effect of using weekly time steps on the estimates, which required a less granular discretization of the incubation period and TOST into weekly intervals. Let w denote the week since the index event (exposure for the incubation period, or symptom onset for the TOST). For the incubation period, we assumed the probability of symptom onset within the same week as exposure ($w = 0$), or beyond week 3 ($w > 3$), to be negligible:

$$f_w = \begin{cases} \dfrac{F(7(w + 0.5)) - F(7(w - 0.5))}{F(7(w_{\max} + 0.5)) - F(7(w_{\min} - 0.5))}, & w = 1,2,3 \\ 0, & \text{otherwise} \end{cases}$$

where F is the cumulative distribution function (CDF) of the incubation period, and $w_{\min} = 1$ and $w_{\max} = 3$. Substituting the fitted parameters into f_w yielded the following probability mass:

$$f_w = \begin{cases} 0.6044, & w = 1 \\ 0.3682, & w = 2 \\ 0.0274, & w = 3 \\ 0, & \text{otherwise} \end{cases}$$

In contrast, for the TOST distribution we assumed that transmission within the same week as symptom onset ($w = 0$) could not be neglected:

$$\lambda_w = \begin{cases} \dfrac{\Lambda(7(w + 0.5)) - \Lambda(7(w - 0.5))}{\Lambda(7(w_{\max} + 0.5))}, & w = 0,1,2,3 \\ 0, & \text{otherwise} \end{cases}$$

where Λ is the CDF of the TOST distribution, $w_{\min} = 0$, and $w_{\max} = 3$; because $\Lambda(7(w_{\min} - 0.5)) = 0$, the lower term of the normalizing denominator vanishes. λ_w yielded the following probability mass:

$$\lambda_w = \begin{cases} 0.3341, & w = 0 \\ 0.6406, & w = 1 \\ 0.0250, & w = 2 \\ 0.0003, & w = 3 \\ 0, & \text{otherwise.} \end{cases}$$

2.3. Transmission dynamics

2.3.1. Renewal process with intrinsic backprojection

To allow the model to account for the effect of control measures, such as case isolation and contact tracing, that reduce transmissibility at the time of exposure, we employed a renewal process with intrinsic backprojection that was described earlier (*7*,*8*). The method assumes transmission to be post-symptomatic, so that the underlying transmission networks contain no closed cycles (see discussion (*9*)). This assumption is likely valid for Ebola viruses.

We modeled several infections j_t on day t that were generated by the cases with symptom onset in the current and preceding days ($t, t-1, \dots, t - t_{\max}^{\text{ost}}$), weighted by the TOST distribution λ_t and scaled by the reproduction number R_t:

$$j_t = R_t \sum_{s=0}^{t_{\max}^{\text{ost}}} c_{t-s}\, \lambda_s$$

Because the symptom onset among new infections is lagged by the incubation period, the expected number of cases c_t on day t was given by:

$$E[c_t] = \sum_{\tau=1}^{t_{\max}} j_{t-\tau}\, f_\tau$$

If we combine last two equations, we get:

$$E[c_t] = \sum_{\tau=1}^{t_{\max}} R_{t-\tau}\, f_\tau \sum_{s=0}^{t_{\max}^{\text{ost}}} c_{t-\tau-s}\, \lambda_s$$

The expected counts can be then linked to the daily incidence through a negative binomial likelihood:

$$c_t \sim \text{NegativeBinomial}(\text{mean} = E[c_t], \text{overdisp.} = \phi)$$

where c_t is the case count on day t, and ϕ is the overdispersion parameter.

Because the data were aggregated to weekly counts, the weekly count is a sum of daily negative binomials

$$c_w \sim \sum_{t \text{ in week } w} \text{NegativeBinomial}(\text{mean} = E[c_t], \text{overdisp.} = \phi)$$

which is a compound distribution that we approximated by a single negative binomial through matching of its first two moments (*12*):

$$c_w \sim \text{NegativeBinomial}(\text{mean} = E[c_w], \text{overdisp.} = \phi_w)$$

$$E[c_w] = \sum_{t \text{ in week } w} E[c_t]\,, \phi_w = \phi \cdot \frac{(E[c_w])^2}{\sum_{t \text{ in week } w}(E[c_t])^2}$$

2.3.2. Adjusting for the effect of control measures

The control measures implemented in week 48 had multiple effects on the transmission dynamics. First, they reduced transmission, resulting in reduction of the reproduction number. Second, they improved case ascertainment, with some fraction of cases before the intervention to be retrospectively added to the final case count. Third, the risk of dying was also reduced due to more efficient case management, e.g., earlier hospitalization has been shown to reduce mortality rates. These factors were added to our model using the following:

1. The decrease in transmission due to interventions was modeled by a stepwise function of time:

$$R_t = \begin{cases} \text{preintervention } R_{\text{eff}}, & t < t_{\text{int}} \\ \text{postintervention } R_{\text{eff}}, & \text{otherwise} \end{cases}$$

where $t_{\text{int}} = 48$ week (in days) and $\text{postintervention } R_{\text{eff}} < \text{preintervention } R_{\text{eff}}$.

2. The case ascertainment rate was also modeled by a two-level stepwise function of time. We supposed that some fraction of cases p is finally reported after retrospective investigation, with the part $(1 - p)$ remaining missing. Specifically, we imposed the following functional form:

$$p_t = \begin{cases} p, & t < t_{\text{int}} \\ 1, & \text{otherwise} \end{cases}$$

3. The cases were assumed to start seeking care or getting hospitalized, resulting in higher chances of survival. This resulted in the reduction in case-fatality rate (CFR) by a multiplicative factor $q < 1$, characterized by week w:

$$q_w = \begin{cases} 1, & w < 47 \text{ week} \\ (1+q)/2, & w = 47 \text{ week} \\ q, & w \geq 48 \text{ week} \end{cases}$$

where we also assumed that the hospitalization could be efficient if the patient is hospitalized within 3 days of their symptom onset (i.e.., during the presumptive "dry" clinical phase of the disease), which was reflected in the reduction in CFR in week 47.

2.4. Model specifications

We evaluated the model performance across a grid of specifications that varied the following five elements:

1. Temporal resolution. We used a daily renewal process, aggregating the daily expected counts to the observed weekly totals through a moment-matched negative binomial approximation (*12*). We also investigated the fit of the data for directly incorporating the weekly intervals into the model.

2. Incubation period distribution. In place of the gamma distribution of the main analysis, we also used a triangular distribution fitted to the published ranges of the incubation period for Bundibugyo virus disease (BVD), and estimated the gamma distribution for Ebola virus disease (EVD) as comparison.

3. Transmission-dynamics model structure. In the semi-mechanistic structure, the observed incidence, adjusted to under-ascertainment, drove the force of infection, as in the main analysis, corresponding to the instantaneous reproduction number (*13*). In the fully mechanistic structure, a latent, self-generating infection process formed the epidemic trajectory, with process noise on the latent infections, corresponding to a state-space renewal model (*14–16*).

4. Within-week allocation (semi-mechanistic models only). The observed weekly cases were distributed across the 7 days of the week either (*i*) uniformly or (*ii*) through a latent simplex of size 7 with a non-informative Dirichlet prior.

5. Process-noise parameterization (fully mechanistic models only). The latent infections followed a normal distribution with the renewal mean (same as in the Cori et al. framework), but under either a variance-to-mean ratio (VTM) or a coefficient of variation (CV) parameterization of the process variance, following the framework developed elsewhere (*15*,*16*).

In total, fitted models included 32 models at daily resolution, covering 4 incubation-period distributions for BVD (triangular, gamma, Weibull and lognormal), 4 model structures (semi-mechanistic with uniform or Dirichlet allocation, and fully mechanistic with VTM or CV process noise), and 2 case-fatality likelihoods (the binomial evaluated at the non-integer completed counts, and its gamma approximation). Three were at weekly resolution with the triangular incubation period (BVD), under the semi-mechanistic (baseline) or the fully mechanistic structure with VTM or CV process noise. Three used the gamma incubation period reported for EVD at daily resolution, under the semi-mechanistic and the two fully mechanistic structures. In addition, we conducted three sensitivity analyses: a time-varied ascertainment rate (6 models), a grid over the partial-ascertainment of both cases and deaths (100 models), and a sweep over the mean TOST (3 models), giving 147 model variations in total.

2.4.1. Semi-mechanistic model

To account for case ascertainment, we introduced the total case count $\tilde{c}_t$ on day t, and modified the double convolution written above to the form:

$$E[\tilde{c}_t] = \sum_{\tau=1}^{t_{\max}} R_{t-\tau}\, f_\tau \sum_{s=0}^{t_{\max}^{\text{ost}}} \tilde{c}_{t-\tau-s}\, \lambda_s$$

linked to the daily case counts through the factor p_t

$$c_t \sim \text{NegativeBinomial}(\text{mean} = p_t E[\tilde{c}_t], \text{overdisp.} = \phi)$$

The remaining fraction $(1 - p_t)$ contributed to the adjusted case counts (*10*):

$$\tilde{c}_t = c_t + (1 - p_t) E[\tilde{c}_t]$$

Thus, $\tilde{c}_t$ was identified recursively starting from $t = 1$ and moving forward in time. The very first count, corresponding to the index case, was assumed to be completely ascertained, so that $\tilde{c}_1 = c_1$.

Because such recursion requires imputation of the daily case counts c_t, that were not observed, we allocated the observed weekly cases across 7 days of the week in two ways: uniformly, $c_{t \text{ in week } w} = c_w/7$; or as a latent allocation, $c_{t \text{ in week } w} = c_w \cdot \Omega_w$, where Ω_w is a simplex of size seven ($\sum_{i=1}^{7} \Omega_{w,i} = 1$) with a symmetric non-informative prior: $\Omega_w \sim \text{Dirichlet}(1,1,1,1,1,1,1)$.

The CFR appeared biased upward as the denominator is smaller for the ascertained cases. We assumed that all deaths are fully ascertained. To adjust for this bias, we used the adjusted weekly case counts $\tilde{c}_w$ in the numerator of the CFR formula and embed the binomial likelihood applied to each week w:

$$d_w \sim \text{Binomial}(\text{size} = \tilde{c}_w, \text{prob.} = q_w\, \text{CFR})$$

Because the value $\tilde{c}_w$ is not integer-valued, we defined its proxy by using the log of the Binomial probability mass function adapted to non-integer values. Specifically, we replaced the log of the binomial coefficient by a combination of log-Gamma functions

$$\log \Pr(d_w) = \log \Gamma(\tilde{c}_w + 1) - \log \Gamma(d_w + 1) - \log \Gamma(\tilde{c}_w - d_w + 1) +$$
$$d_w \log(q_w\, \text{CFR}) + (\tilde{c}_w - d_w) \log(1 - q_w\, \text{CFR}), \quad \text{when } d_w > 0$$

However, when the death count on a given week w is zero, we used the following expression:

$$\log \Pr(d_w) = \tilde{c}_w \log(1 - q_w\, \text{CFR}), \quad \text{when } d_w = 0$$

We note that the used proxy defines an improper probability density, since normalization to one does not hold. We also considered the approximation of the $\log \Pr(d_w)$ using a moment-matched Gamma distribution as sensitivity analysis (*11*).

2.4.2. Fully mechanistic model

We also modeled transmission dynamics with a fully mechanistic approach incorporating process noise, in two parameterizations following the framework of *R* package *epidemia* (*16*). The outbreak timeline was composed of two periods: a pre-observation period, starting 14 days before the day 1 (the characteristic time scale of the generation time for Ebola virus), when initial infections were seeded, and an observation period, starting from day 1.

During the pre-observation period, infections $j_{-13:0}$ were seeded at constant rate $j_{-t} = j_{\text{seed}}$ for all $t \in \{13, \ldots, 0\}$ with a hierarchical prior (*15*,*16*):

$$j_{\text{seed}} \sim \text{Exponential}(\text{rate} = \tau^{-1})$$

$$\tau \sim \text{Exponential}(\text{rate} = \nu)$$

where $\nu > 0$ is a rate hyperparameter given by a prior Half-Normal(mean = 0, SD = 2).

The latent daily infections were centered at the expected value μ_t:

$$\mu_t = R_{w(t)} \sum_{s=0}^{d_{\max}^{\text{ost}}} E[\tilde{c}_{t-s}]\, \lambda_s, \qquad E[\tilde{c}_t] = \sum_{s=1}^{d_{\max}} j_{t-s}\, f_s$$

Under the VTM parameterization, the process variance was proportional to the mean

$$j_t = \max(\mu_t + \epsilon_t \cdot \sqrt{\delta \cdot \mu_t}, 0), \qquad \epsilon_t \sim \text{Normal}(\text{mean} = 0, \text{SD} = 1)$$

with dispersion (VTM ratio) δ, given by a prior Exponential(rate = 1).

Under the CV parameterization the variance was proportional to the squared mean

$$j_t = \max(\mu_t + \epsilon_t \cdot \text{CV} \cdot \mu_t, 0), \qquad \epsilon_t \sim \text{Normal}(\text{mean} = 0, \text{SD} = 1)$$

with constant CV, given by a prior Half-Normal(mean = 0, SD = 0.5).

2.4.3. Gamma approximation of the binomial for estimating CFR

The CFR appeared biased upward as the denominator is smaller for the ascertained cases. We assumed that all deaths are fully ascertained. To adjust for this bias, we used the adjusted weekly case counts $\tilde{c}_w$ in the numerator of the CFR formula and embed the binomial likelihood applied to each week w:

$$d_w \sim \text{Binomial}(\text{size} = \tilde{c}_w, \text{prob.} = q_w\, \text{CFR})$$

Because the value $\tilde{c}_w$ is not integer-valued, we apply the following two-step mathematical trick. First, we rewrite the binomial likelihood to the negative binomial likelihood, but respectively to the case counts:

$$\tilde{c}_w - d_w \sim \text{NegBinomial}(\text{size} = d_w, \text{prob.} = q_w\, \text{CFR})$$

Second, we approximate this likelihood using the gamma distribution with matched first and second moments (*11*):

$$\tilde{c}_w - d_w \sim \text{Gamma}(\text{shape} = d_w \cdot (1 - q_w\,\text{CFR}), \text{rate} = q_w\,\text{CFR}), \quad \text{when } d_w > 0$$

However, when the death count on a given week w is zero, the gamma distribution is not defined. To handle this case, we use the log-likelihood of the binomial distribution when $d_w = 0$:

$$\log \Pr(\tilde{c}_w) = \tilde{c}_w \log(1 - q_w\,\text{CFR}), \quad \text{when } d_w = 0$$

2.4.4. Partial ascertainment of both cases and deaths

For the sensitivity analysis, we also assumed that the interventions on week 48 could not lead to complete ascertainment of cases, leaving some of their fraction under-ascertained:

$$p_t = \begin{cases} p, & t < t_{\text{int}} \\ (1-\varepsilon) \cdot p + \varepsilon, & \text{otherwise} \end{cases}$$

where ε is the reduction in under-ascertainment with $\varepsilon = 1$ representing the baseline scenario. We also considered when cases in the preintervention period would also be under-ascertained. The overall under-ascertainment rate of deaths was modeled by a variable θ. We assumed that some cases among unascertained could have died

$$\tilde{d}_t = d_t + (1-\theta) \cdot q_w\,\text{CFR} \cdot (1 - p_t) E[\tilde{c}_t]$$

Then the weekly death counts are defined as follows

$$\tilde{d}_w = d_w + (1-\theta) \cdot q_w\,\text{CFR} \cdot \sum_{t\ in\ week\ w} (1 - p_t) E[\tilde{c}_t]$$

with the Binomial likelihood written in the form

$$\tilde{d}_w \sim \text{Binomial}(\text{size} = \tilde{c}_w, \text{prob.} = q_w\,\text{CFR})$$

resulting in the following log-likelihood adapted to non-integer $\tilde{d}_w$ and $\tilde{c}_w$:

$$\log \Pr(\tilde{d}_w) = \log \Gamma(\tilde{c}_w + 1) - \log \Gamma(\tilde{d}_w + 1) - \log \Gamma(\tilde{c}_w - \tilde{d}_w + 1) +$$

$$\tilde{d}_w \log(q_w\,\text{CFR}) + (\tilde{c}_w - \tilde{d}_w) \log(1 - q_w\,\text{CFR}), \quad \text{when } \tilde{d}_w > 0$$

and

$$\log \Pr(\tilde{d}_w) = \tilde{c}_w \log(1 - q_w \,\text{CFR}), \quad \text{when } \tilde{d}_w = 0$$

2.4.5. Choice of priors

The key model parameters followed weakly informative priors in the simulations

$$\log(\text{preintervention } R_{\text{eff}}) \sim \text{Normal}(\text{mean} = \log(1.5), \text{SD} = 1)$$

$$\log(\text{postintervention } R_{\text{eff}}) \sim \text{Normal}(\text{mean} = \log(1.5), \text{SD} = 1)$$

with imposed ordering: preintervention $R_{\text{eff}} >$ postintervention R_{eff};

$$\phi^{-1/2} \sim \text{Exponential}(\text{rate} = 1)$$

$$p \sim \text{Beta}(\text{shape}_1 = 6, \text{shape}_2 = 4)$$

$$\text{logit}(\text{preintervention CFR}) \sim \text{Normal}(\text{mean} = 0, \text{SD} = 1.5)$$

$$\text{logit}(\text{postintervention CFR}) \sim \text{Normal}(\text{mean} = 0, \text{SD} = 1.5)$$

similarly with imposed ordering: preintervention CFR > postintervention CFR.

For fully mechanistic model, we defined

$$\nu \sim \text{Half-Normal}(\text{mean} = 0, \text{SD} = 2)$$

whereas, depending on parameterization,

$$\delta \sim \text{Exponential}(\text{rate} = 1) \text{ or CV} \sim \text{Half-Normal}(\text{mean} = 0, \text{SD} = 0.5)$$

3. Sensitivity analysis

3.1. Model variations

Across all model specifications, R_{eff} was qualitatively robust, with the posterior mean of the preintervention R_{eff} above one declining to a postintervention mean below one. However, the posterior mean depended on model structure. Other key parameters, including pre- and postintervention CFRs, and the under-ascertainment rate, were insensitive to the transmission-model structure and varied only with the choice of case-fatality likelihood (see below).

The temporal resolution (daily or weekly modeling of incidence) and incubation period (gamma, Weibull, lognormal, or triangular distribution) had limited effect on the R_{eff} estimates (Appendix Table 1). The gamma, Weibull and lognormal distributions yielded nearly identical

estimates. Varying the within-week allocation of weekly case totals, either by uniform distribution or by the latent Dirichlet posterior, also produced nearly identical estimates, implying that the data do not resolve sub-weekly timing of cases. The only configuration that noticeably affected the estimates was the transition from the semi-mechanistic to the fully mechanistic model. Among the daily models, the semi-mechanistic estimates of the preintervention mean R_{eff} varied between 1.54 and 1.68, while the fully mechanistic estimates were lower, between 1.13 and 1.28. This gap was systematic: the semi-mechanistic model was driven by the observed weekly incidence and represented stochasticity through the observation noise, whereas the fully mechanistic model tracked a smooth self-exciting trajectory generated from a seed block of initial infections (*15*). The difference lay in how the two models treated the peak observed in week 48. The semi-mechanistic model attributed the peak to transmission from the preceding cases, while the fully mechanistic model absorbed the peak in the process noise. Additionally, process noise was identifiable only within the fully mechanistic approach. Adding process noise to the semi-mechanistic model made R_{eff} confounded with the noise scale, and the MCMC simulations failed to converge.

Because conditional renewal estimation may over-estimate R_{eff} during early stages of the outbreak when first generations are largely under-reported (*17*), we defined the fully mechanistic model with an explicit pre-observation seed block varying its length (single-day, 7 days, or 14 days). For varied length of a seed block, the preintervention R_{eff} changed only slightly. For example, the fully mechanistic model with the CV parameterization and incubation period given by gamma distribution yielded posterior mean R_{eff} of 1.31, 1.28, and 1.28, for seed blocks of these lengths, respectively. Estimated total number of seed infections was ≈2 across all fully mechanistic models. Hence, the hypothesis of a large unobserved case count preceding the outbreak appears unlikely.

Using the gamma approximation to the binomial likelihood instead of the (quasi-) binomial evaluated at non-integers raised the preintervention CFR by 3 to 4 percentage points and lowered the estimated under-ascertainment (Appendix Table 2). At weekly resolution, the semi-mechanistic estimate was unchanged (1.59 versus 1.55 at daily resolution), whereas the fully mechanistic estimates were less precise, with 95% CrIs much wider than at daily resolution (Appendix Table 3). Incorporating the EVD incubation period elevated the preintervention mean

R_{eff} from 1.55 to 1.71 and reduced the postintervention mean R_{eff} from 0.50 to 0.37 (Appendix Table 4). Varying the mean TOST between 3 and 6 days moved the preintervention mean R_{eff} from 1.40 to 1.62 (Appendix Table 5).

In summary, the baseline model represented a standard, widely used instantaneous R_{eff} based on a modified Cori et al. method (*13,7*), and gave pre- and postintervention mean R_{eff} of 1.55 and 0.50, respectively. The other models were considered as supplementary and confirmatory, resulting in an overall range from 1.13 to 1.74 for preintervention mean R_{eff} and 0.28 to 0.58 for the postintervention mean R_{eff}.

3.2. Time-varied case under-ascertainment

As part of the sensitivity analysis, we assumed that some fraction of cases that occurred long before the intervention would be more likely to be missed compared to cases closer to the time of intervention. This reflects the documented response: village health teams conducted active searches through daily door-to-door visits in the villages, and investigation teams searched for case-patients in health facilities and communities and retrospectively reviewed hospital records (*1*). Case-finding after week 48 therefore also recovered cases with earlier onset, but with diminishing efficiency back in time. We investigated the following functional form for the ascertainment rate $p(t)$:

$$p(t) = \check{p} + (1 - \check{p}) \cdot \exp(-(t_{\text{int}} - t)/\tau_{\text{rep}}), \quad \text{where } t \leq t_{\text{int}}$$

and $p(t) = 1$ for $t > t_{\text{int}}$, when all cases are ascertained. Here τ_{rep} is the characteristic time over which retrospective case investigation remained effective, so that ascertainment is complete at the time of intervention but exponentially decays backward in time toward $\check{p}$. Two limiting cases cover the model specification considered elsewhere in the Appendix: as τ_{rep} approaches zero, $p(t)$ tends to $\check{p}$ in step-like manner, recovering our baseline model; as τ_{rep} becomes large, $p(t)$ tends to one throughout the outbreak, corresponding to the complete ascertainment over the whole period. Larger τ_{rep} therefore imply that retrospective case-finding reached further into the past, giving higher ascertainment and fewer missed cases. We fixed τ_{rep} in our simulations at 2, 4, and 8 weeks. The results of simulations did not show any substantial difference in the estimated parameters when the ascertainment rate is time-varying (Appendix Figure 2, Appendix Figure 3).

3.3. Partial ascertainment of both cases and deaths

Because ε and θ are unidentifiable, we conducted a series of simulations on a grid of values $0 \leq \varepsilon, \theta \leq 1$. $\varepsilon = 0$ implied that no changes in ascertainment could be seen before and after the interventions. $\varepsilon = 1$, which corresponds to the baseline, implied that only cases in the preintervention period remained under-ascertained. On the other hand, values of θ close to zero implied that only a small fraction of deaths were reported in the preintervention period, while $\theta = 1$ implied that all deaths were similarly ascertained in both pre- and postintervention periods. The simulations showed that variation in both ε and θ did not affect the robustness of reported estimates: the posterior means for the preintervention mean R_{eff} varied 1.51–1.65 (baseline model estimate was 1.55), the postintervention mean R_{eff} varied 0.43–0.50 (baseline: 0.50), preintervention CFR varied 31.4%–39.2% (baseline: 31.4%), postintervention CFR varied 24.9%–28.7% (baseline: 25.1%), overall under-ascertainment of cases 14.9%–24.8% (baseline: 14.9%), overall under-ascertainment of deaths 0%–25.1% (baseline: 0%) (Appendix Figure 4).

4. Technical details

Model fitting was performed in a Bayesian framework using Markov chain Monte Carlo (MCMC) sampling. We used the No-U-Turn Sampler (NUTS) implemented in the Stan software for efficient sampling from the posterior distribution of the parameters (https://mc-stan.org). The model was run with 5 parallel chains, each consisting of 15,000 iterations, with the first 2,500 iterations discarded as burn-in. Convergence of the chains was assessed using the $\hat{R}$-statistic, with values below 1.01 indicating satisfactory convergence.

5. Model diagnostics

We evaluated the fit of the baseline model with two checks. First, the posterior distributions deviated substantially from their priors, indicating that parameters were informed by the data rather than by the priors (Appendix Figure 5). Second, a posterior predictive check compared the observed weekly case counts with those predicted by the model (Appendix Figure 6).

Although the renewal process did not adequately capture the surge in cases in week 48, most likely due to superspreading (Appendix Figure 6A), this was the only week where observed

count fell outside the 95% posterior predictive interval. The observed counts of all remaining weeks were covered (Appendix Figure 6B).

Overall, across all 47 main models (Appendix Tables 1–5, Appendix Figures 2–3), $\hat{R}$ did not exceed 1.01 and the minimum bulk effective sample size (ESS) was >10,000. Only 22 divergent transitions were identified among nearly 3 million posterior draws.

6. AI disclosure

During the preparation of this work, A.R.A. used Claude Code (Anthropic), powered by the Claude Opus 4.8 large language model, to assist with code development and English-language editing of the manuscript. All authors reviewed and edited all output, and take full responsibility for the accuracy and integrity of the work. The study design, analysis, interpretation, and conclusions are those of the authors, and AI tools were not used to generate or interpret the findings. The code scripts and all accompanying material are available at https://github.com/aakhmetz/Bundibugyo2007-transmission.

7. Study limitations

Our study has several limitations. First, it is based on a single, small outbreak (110 cases, 37 deaths) whose weekly counts were digitized from a published figure (*1*) rather than available from linelist data. The small number of postintervention weeks resulted in wider CrIs for the postintervention reproduction number. These estimates may not be generalizable to a much larger 2026 outbreak. Second, the lack of linelist data was also reflected in the absence of BDBV-specific estimates of epidemiologic time intervals, such as incubation period and time from onset of symptoms to transmission (TOST), generation time or serial interval. We used the TOST estimates specific to Ebola virus (*5*), which could differ from BDBV-specific estimates and result in misspecification. Third, our model reproduces only the mean (expected) transmission dynamics, while largely underestimating a spike of cases in week 48. The latter was likely due to superspreading and could not adequately be captured by the renewal process. To our knowledge, superspreading during this outbreak has not been documented previously (*1*,*18*,*19*,*20*), and we were therefore unable to model it in greater detail. Fourth, our model incorporated the intervention by rigid step functions for the transmission, case ascertainment, and

mortality. However, the true changes were likely more gradual, and the concurrent declines in transmission and mortality could not be fully disentangled from other factors such as behavioral change or population dynamics. Fifth, we assumed that deaths were completely ascertained, while only non-fatal cases were missed before the intervention. However, some deaths could be still missed due to reluctance for post-mortem detection or other factors. This would bias the CFR estimate downward with under-ascertainment being only weakly identified. Finally, our modeling approach rests on two assumptions: the intrinsic backprojection assumes post-symptomatic transmission with no closed transmission cycles (*9*), which is a reasonable assumption for Ebola viruses, and the non-integer adjusted case counts are used in estimation of the CFR.

References

1. Wamala JF, Lukwago L, Malimbo M, Nguku P, Yoti Z, Musenero M, et al. Ebola hemorrhagic fever associated with novel virus strain, Uganda, 2007–2008. Emerg Infect Dis. 2010;16:1087–92. PubMed https://doi.org/10.3201/eid1607.091525

2. Van Kerkhove MD, Bento AI, Mills HL, Ferguson NM, Donnelly CA. A review of epidemiological parameters from Ebola outbreaks to inform early public health decision-making. Sci Data. 2015;2:150019. PubMed https://doi.org/10.1038/sdata.2015.19

3. UK Health Security Agency. Ebola disease outbreak in the Democratic Republic of the Congo and Uganda (Bundibugyo virus) [cited 2026 Jul 20]. https://www.gov.uk/guidance/ebola-disease-outbreak-in-the-democratic-republic-of-the-congo-and-uganda-bundibugyo-virus

4. Aylward B, Barboza P, Bawo L, Bertherat E, Bilivogui P, Blake I, et al.; WHO Ebola Response Team. Ebola virus disease in West Africa—the first 9 months of the epidemic and forward projections. N Engl J Med. 2014;371:1481–95. PubMed https://doi.org/10.1056/NEJMoa1411100

5. Yamin D, Gertler S, Ndeffo-Mbah ML, Skrip LA, Fallah M, Nyenswah TG, et al. Effect of Ebola progression on transmission and control in Liberia. Ann Intern Med. 2015;162:11–7. PubMed https://doi.org/10.7326/M14-2255

6. White L. Key Parameters in Infectious Disease Epidemiology. Ch. 5 in: Handbook of infectious disease data analysis. Eds. Held L, Hens N, O'Neill P, Wallinga J. *CRC Press*. 2020.

7. Nakajo K, Nishiura H. Estimation of R(t) based on illness onset data: an analysis of 1907–1908 smallpox epidemic in Tokyo. Epidemics. 2022;38:100545. PubMed https://doi.org/10.1016/j.epidem.2022.100545

8. Akhmetzhanov AR, Cheng HY, Linton NM, Ponce L, Jian SW, Lin HH. Transmission dynamics and effectiveness of control measures during COVID-19 surge, Taiwan, April–August 2021. Emerg Infect Dis. 2022;28:2051–9. PubMed https://doi.org/10.3201/eid2810.220456

9. Bacallado S, Zhao Q, Ju N. Letter to the editor: generation interval for COVID-19 based on symptom onset data. Euro Surveill. 2020;25:2001381. PubMed https://doi.org/10.2807/1560-7917.ES.2020.25.29.2001381

10. Beesley LJ, Osthus D, Del Valle SY. Addressing delayed case reporting in infectious disease forecast modeling. PLOS Comput Biol. 2022;18:e1010115. PubMed https://doi.org/10.1371/journal.pcbi.1010115

11. Li M, Dushoff J, Bolker BM. Fitting mechanistic epidemic models to data: a comparison of simple Markov chain Monte Carlo approaches. Stat Methods Med Res. 2018;27:1956–67. PubMed https://doi.org/10.1177/0962280217747054

12. Modrák M. Approximate densities for sums of variables: negative binomials and saddlepoint [cited 2026 Jul 20]. https://www.martinmodrak.cz/2019/06/20/approximate-densities-for-sums-of-variables-negative-binomials-and-saddlepoint/

13. Cori A, Ferguson NM, Fraser C, Cauchemez S. A new framework and software to estimate time-varying reproduction numbers during epidemics. Am J Epidemiol. 2013;178:1505–12. PubMed https://doi.org/10.1093/aje/kwt133

14. Flaxman S, Mishra S, Gandy A, Unwin HJT, Mellan TA, Coupland H, et al.; Imperial College COVID-19 Response Team. Estimating the effects of non-pharmaceutical interventions on COVID-19 in Europe. Nature. 2020;584:257–61. PubMed https://doi.org/10.1038/s41586-020-2405-7

15. Scott JA, Gandy A, Mishra S, Bhatt S, Flaxman S, Unwin HJ, et al. Epidemia: an R package for semi-mechanistic bayesian modelling of infectious diseases using point processes. *arXiv preprint.* 2021;2110.12461. https://arxiv.org/abs/2110.12461

16. Scott JA, Gandy A, Mishra S, Unwin J, Flaxman S, Bhatt S. epidemia: Modeling of Epidemics using Hierarchical Bayesian Models. R package version 1.0.0. 2020. https://imperialcollegelondon.github.io/epidemia

17. Brizzi A, O'Driscoll M, Dorigatti I. Refining reproduction number estimates to account for unobserved generations of infection in emerging epidemics. Clin Infect Dis. 2022;75:e114–21. PubMed https://doi.org/10.1093/cid/ciac138

18. Towner JS, Sealy TK, Khristova ML, Albariño CG, Conlan S, Reeder SA, et al. Newly discovered ebola virus associated with hemorrhagic fever outbreak in Uganda. PLoS Pathog. 2008;4:e1000212. PubMed https://doi.org/10.1371/journal.ppat.1000212

19. MacNeil A, Farnon EC, Wamala J, Okware S, Cannon DL, Reed Z, et al. Proportion of deaths and clinical features in Bundibugyo Ebola virus infection, Uganda. Emerg Infect Dis. 2010;16:1969–72. PubMed https://doi.org/10.3201/eid1612.100627

20. Clark DV, Kibuuka H, Millard M, Wakabi S, Lukwago L, Taylor A, et al. Long-term sequelae after Ebola virus disease in Bundibugyo, Uganda: a retrospective cohort study. Lancet Infect Dis. 2015;15:905–12. **PMID 25910637**

Appendix Table 1. Parameter estimates derived from daily models with the BVD incubation period modeled by gamma, Weibull, lognormal or triangular distributions. Values are posterior means, with 95% credible intervals in parentheses. Cases in the postintervention period were assumed to be fully ascertained. Overall under-ascertainment is defined as the estimated number of missed cases divided by the estimated total over the whole outbreak.

Incubation period	Preintervention, R_e	Postintervention, R_e	Preintervention CFR, %	Postintervention CFR, %	Preintervention under-ascertainment, %	Overall under-ascertainment, %
Semi-mechanistic (uniform allocation)						
Gamma (baseline)	1.55 (1.02-2.36)	0.50 (0.17-1.15)	31.4 (21.8-42.8)	25.1 (16.3-34.6)	19.3 (5.9-38.2)	14.9 (2.7-31.7)
Weibull	1.55 (1.02-2.37)	0.51 (0.17-1.16)	31.2 (21.6-42.6)	25.0 (16.3-34.5)	19.6 (6.0-38.5)	15.2 (3.5-32.5)
Lognormal	1.55 (1.02-2.36)	0.49 (0.16-1.13)	31.5 (21.7-42.9)	25.1 (16.2-34.8)	19.2 (5.7-38.0)	14.7 (2.7-31.7)
Triangular	1.68 (1.11-2.58)	0.41 (0.14-1.02)	31.3 (21.8-42.7)	25.0 (16.3-34.5)	18.8 (5.8-36.6)	15.1 (3.5-31.7)
Semi-mechanistic (Dirichlet prior)						
Gamma	1.54 (1.01-2.34)	0.50 (0.17-1.14)	31.5 (21.8-42.9)	25.1 (16.3-34.6)	19.2 (5.8-38.0)	14.7 (2.7-31.7)
Weibull	1.54 (1.02-2.35)	0.51 (0.17-1.15)	31.3 (21.7-42.8)	25.0 (16.3-34.5)	19.6 (5.9-38.4)	15.0 (2.7-32.1)
Lognormal	1.54 (1.02-2.35)	0.49 (0.17-1.13)	31.5 (21.9-43.0)	25.2 (16.4-34.8)	19.0 (5.6-37.7)	14.5 (2.7-31.7)
Triangular	1.67 (1.10-2.57)	0.42 (0.14-1.03)	31.3 (21.7-42.6)	25.0 (16.2-34.4)	18.9 (5.9-36.9)	15.2 (3.5-31.7)
Fully mechanistic (VTM)						
Gamma	1.14 (0.75-1.43)	0.36 (0.13-0.68)	31.6 (21.4-43.4)	25.1 (16.2-34.8)	22.4 (6.4-44.8)	14.7 (2.7-32.5)
Weibull	1.13 (0.74-1.42)	0.37 (0.14-0.68)	31.6 (21.4-43.5)	25.0 (16.2-34.7)	22.5 (6.5-45.0)	14.8 (2.7-32.9)
Lognormal	1.14 (0.74-1.43)	0.35 (0.13-0.68)	31.5 (21.4-43.3)	25.0 (16.2-34.7)	22.4 (6.5-44.8)	14.8 (2.7-32.9)
Triangular	1.18 (0.77-1.48)	0.32 (0.11-0.64)	31.7 (21.5-43.4)	25.2 (16.3-34.9)	22.3 (6.5-44.5)	14.5 (2.7-32.1)
Fully mechanistic (CV)						
Gamma	1.24 (1.04-1.45)	0.39 (0.19-0.66)	31.7 (21.4-43.5)	25.1 (16.1-34.9)	21.7 (6.2-44.3)	14.5 (2.7-32.9)
Weibull	1.24 (1.04-1.45)	0.40 (0.20-0.67)	31.7 (21.4-43.6)	25.1 (16.1-34.9)	21.7 (6.2-44.2)	14.5 (2.7-32.5)
Lognormal	1.25 (1.05-1.45)	0.38 (0.18-0.66)	31.7 (21.5-43.5)	25.1 (16.2-34.8)	21.7 (6.2-43.8)	14.5 (2.7-32.5)
Triangular	1.28 (1.06-1.51)	0.34 (0.16-0.61)	31.8 (21.5-43.6)	25.2 (16.3-34.9)	21.8 (6.2-44.0)	14.3 (2.7-32.1)

R_e, effective reproduction number; CFR, case-fatality rate; VTM, variance-to-mean parameterization; CV, coefficient of variation parameterization; BVD, Bundibugyo virus disease.

Appendix Table 1. Changes in parameter estimates when the Gamma approximation of the Binomial likelihood for calculating CFR was used instead of quasi-Binomial approach (Appendix Table 1). Values are posterior means, with 95% credible intervals in parentheses. Cases in the postintervention period were assumed to be fully ascertained. Overall under-ascertainment is defined as the estimated number of missed cases divided by the estimated total over the whole outbreak.

Incubation period	Preintervention, R_e	Postintervention, R_e	Preintervention CFR, %	Postintervention CFR, %	Preintervention under-ascertainment, %	Overall under-ascertainment, %
Semi-mechanistic (uniform allocation)						
Gamma	1.58 (0.99-2.50)	0.54 (0.18-1.22)	34.5 (24.5-46.2)	26.1 (17.0-35.9)	12.4 (3.2-27.2)	9.4 (0.9-22.5)
Weibull	1.59 (0.99-2.51)	0.55 (0.18-1.24)	34.4 (24.5-46.1)	26.1 (17.0-35.7)	12.6 (3.2-27.6)	9.6 (0.9-23.1)
Lognormal	1.58 (0.98-2.49)	0.53 (0.17-1.21)	34.6 (24.6-46.4)	26.1 (17.0-35.9)	12.3 (3.1-27.0)	9.2 (0.9-22.0)
Triangular	1.74 (1.10-2.76)	0.44 (0.14-1.06)	34.3 (24.4-46.0)	26.0 (17.0-35.7)	12.4 (3.3-26.9)	10.0 (1.8-23.6)
Semi-mechanistic (Dirichlet prior)						
Gamma	1.57 (0.98-2.47)	0.54 (0.18-1.22)	34.6 (24.6-46.4)	26.2 (17.1-35.9)	12.3 (3.1-27.0)	9.2 (0.9-22.5)
Weibull	1.57 (0.98-2.49)	0.55 (0.18-1.23)	34.5 (24.4-46.3)	26.1 (17.0-35.9)	12.6 (3.3-27.4)	9.5 (0.9-22.5)

Incubation period	Preintervention, R_e	Postintervention, R_e	Preintervention CFR, %	Postintervention CFR, %	Preintervention under-ascertainment, %	Overall under-ascertainment, %
Lognormal	1.57 (0.98-2.48)	0.53 (0.17-1.19)	34.7 (24.6-46.6)	26.2 (17.0-35.9)	12.2 (3.1-26.8)	9.1 (0.9-22.0)
Triangular	1.74 (1.09-2.75)	0.44 (0.14-1.10)	34.3 (24.5-45.9)	26.0 (16.8-35.7)	12.4 (3.3-26.8)	10.0 (1.8-23.1)
Fully mechanistic (VTM)						
Gamma	1.16 (0.74-1.47)	0.37 (0.14-0.70)	35.0 (24.8-46.8)	26.2 (17.1-36.0)	12.5 (3.1-27.8)	7.9 (0.9-19.7)
Weibull	1.16 (0.75-1.46)	0.38 (0.14-0.70)	35.0 (24.9-46.7)	26.2 (17.0-36.0)	12.6 (3.2-27.9)	7.9 (0.9-19.7)
Lognormal	1.17 (0.75-1.47)	0.36 (0.13-0.69)	35.0 (24.9-46.9)	26.3 (17.1-36.1)	12.5 (3.1-27.9)	7.9 (0.9-19.7)
Triangular	1.21 (0.78-1.53)	0.32 (0.12-0.65)	35.0 (24.9-46.8)	26.2 (17.0-36.0)	12.6 (3.2-28.0)	7.8 (0.9-19.1)
Fully mechanistic (CV)						
Gamma	1.28 (1.08-1.49)	0.39 (0.19-0.68)	35.0 (25.0-46.9)	26.2 (17.1-36.0)	12.3 (3.1-27.3)	7.8 (0.9-19.7)
Weibull	1.28 (1.08-1.49)	0.40 (0.20-0.68)	35.0 (24.9-47.0)	26.2 (17.0-35.9)	12.3 (3.1-27.5)	7.8 (0.9-19.7)
Lognormal	1.28 (1.08-1.49)	0.39 (0.18-0.67)	35.0 (25.0-46.9)	26.3 (17.1-36.0)	12.3 (3.1-27.6)	7.9 (0.9-19.7)
Triangular	1.32 (1.10-1.56)	0.34 (0.16-0.61)	35.0 (24.9-46.9)	26.2 (17.1-36.0)	12.4 (3.1-27.6)	7.8 (0.9-19.1)

R_e, effective reproduction number; CFR, case-fatality rate; VTM, variance-to-mean parameterization; CV, coefficient of variation parameterization.

Appendix Table 2. Changes in parameter estimates when the employed model was at weekly scale and the BVD incubation period was given by a triangular distribution. Values are posterior means, with 95% credible intervals in parentheses. Cases in the postintervention period were assumed to be fully ascertained. Overall under-ascertainment is defined as the estimated number of missed cases divided by the estimated total over the whole outbreak.

**Model**	**Preintervention, R_e**	**Postintervention, R_e**	**Preintervention CFR, %**	**Postintervention CFR, %**	**Preintervention under-ascertainment, %**	**Overall under-ascertainment, %**
Semi-mechanistic (uniform allocation)	1.59 (1.05-2.45)	0.42 (0.14-1.03)	31.3 (21.8-42.6)	25.0 (16.3-34.5)	19.6 (6.1-38.1)	15.0 (3.5-32.1)
Fully mechanistic (VTM)	1.34 (1.01-1.79)	0.37 (0.14-0.74)	32.2 (22.0-43.9)	25.4 (16.3-35.1)	21.5 (6.1-43.2)	13.5 (2.7-30.8)
Fully mechanistic (CV)	1.61 (1.09-3.01)	0.35 (0.14-0.74)	31.7 (21.4-43.7)	25.1 (16.2-34.8)	22.8 (6.5-45.7)	14.4 (2.7-32.5)

R_e, effective reproduction number; CFR, case-fatality rate; VTM, variance-to-mean parameterization; CV, coefficient of variation parameterization; BVD, Bundibugyo virus disease.

Appendix Table 3. Changes in parameter estimates when the incubation period was modeled by a gamma distribution fitted to EVD. Values are posterior means, with 95% credible intervals in parentheses. Cases in the postintervention period were assumed to be fully ascertained. Overall under-ascertainment is defined as the estimated number of missed cases divided by the estimated total over the whole outbreak.

Model	Preintervention, R_e	Postintervention, R_e	Preintervention CFR, %	Postintervention CFR, %	Preintervention under-ascertainment, %	Overall under-ascertainment, %
Semi-mechanistic (uniform allocation)	1.71 (1.13-2.61)	0.37 (0.11-0.95)	31.8 (22.1-43.2)	25.3 (16.4-34.8)	17.5 (5.3-34.8)	13.9 (2.7-29.9)
Fully mechanistic (VTM)	1.21 (0.80-1.53)	0.28 (0.08-0.60)	31.8 (21.7-43.6)	25.1 (16.2-34.8)	22.1 (6.5-44.1)	14.2 (2.7-31.7)
Fully mechanistic (CV)	1.31 (1.07-1.56)	0.29 (0.12-0.57)	31.9 (21.6-43.6)	25.2 (16.3-34.9)	21.8 (6.3-43.8)	14.1 (2.7-31.7)

R_e, effective reproduction number; CFR, case-fatality rate; VTM, variance-to-mean parameterization; CV, coefficient of variation parameterization; EVD, Ebola virus disease.

Appendix Table 4. Changes in parameter estimates when the mean time from onset to transmission (TOST) was varied within the baseline semi-mechanistic model. Values are posterior means, with 95% credible intervals in parentheses. Cases in the postintervention period were assumed to be fully ascertained. Overall under-ascertainment is defined as the estimated number of missed cases divided by the estimated total over the whole outbreak.

Mean TOST, days	Preintervention, R_e	Postintervention, R_e	Preintervention CFR, %	Postintervention CFR, %	Preintervention under-ascertainment, %	Overall under-ascertainment, %
3	1.40 (0.93-2.11)	0.58 (0.21-1.18)	32.1 (22.2-43.6)	25.5 (16.6-35.0)	18.6 (5.4-37.4)	13.3 (2.7-30.4)
4.5	1.53 (1.01-2.32)	0.51 (0.17-1.15)	31.4 (21.7-43.0)	25.1 (16.3-34.6)	19.3 (5.8-38.1)	14.7 (2.7-31.7)
6	1.62 (1.06-2.49)	0.46 (0.16-1.09)	31.3 (21.7-42.6)	25.0 (16.2-34.4)	19.2 (5.9-37.8)	15.2 (3.5-32.1)

R_e, effective reproduction number; CFR, case-fatality rate.

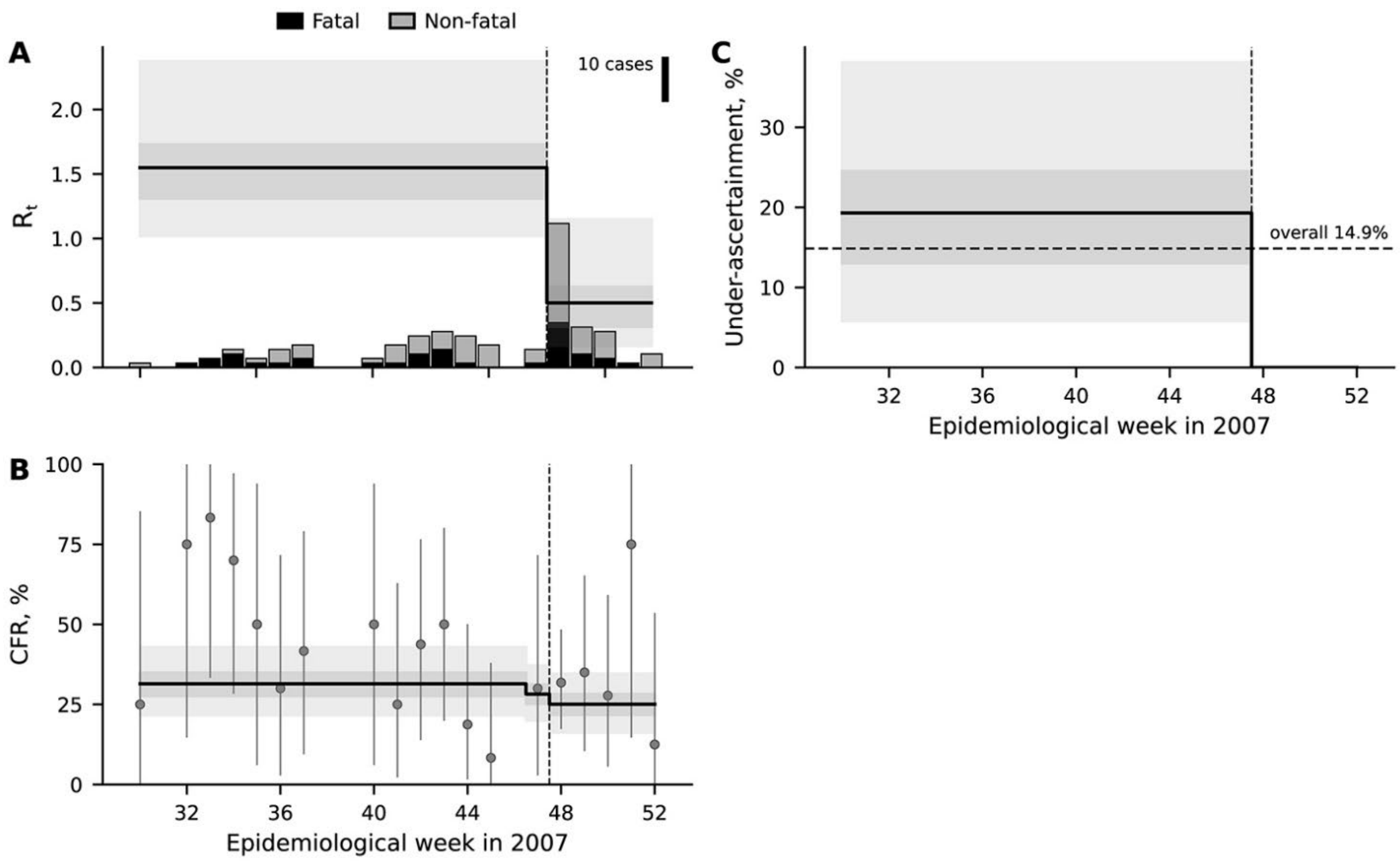

Appendix Figure 1. Model fit to the data from the first Bundibugyo outbreak in Uganda, 2007. (A) Effective reproduction number (R_{eff}) with the reported weekly incidence of fatal and non-fatal cases (legend) shown in the background. (B) Case-fatality rate (CFR) with weekly observed CFR estimates shown in the background; gray dots represent the posterior means from a β-binomial model with Jeffreys' prior $\text{Beta}(0.5{,}0.5)$, and whiskers represent the corresponding 95% CrIs. (C) Under-ascertainment of cases during the outbreak. In all panels, solid lines represent posterior means of the main model; light and dark shaded areas indicate 95% and 50% (interquartile) CrIs; the vertical dashed line represents the week of control measures implementation.

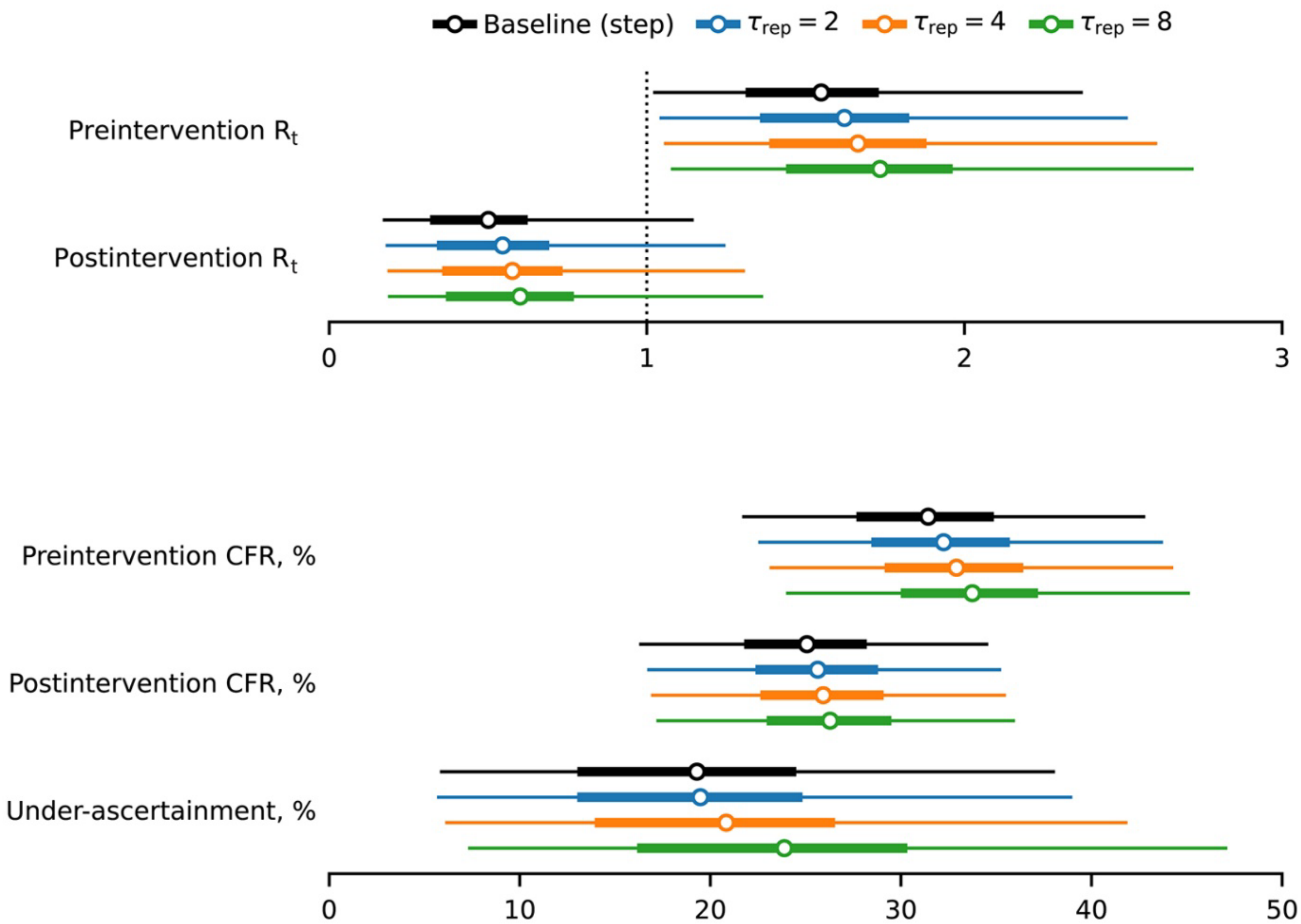

Appendix Figure 2. Sensitivity analysis of main model parameters on the time-varying case under-ascertainment parameter, τ_{rep}. R_{eff}, effective reproduction number; CFR, case-fatality rate.

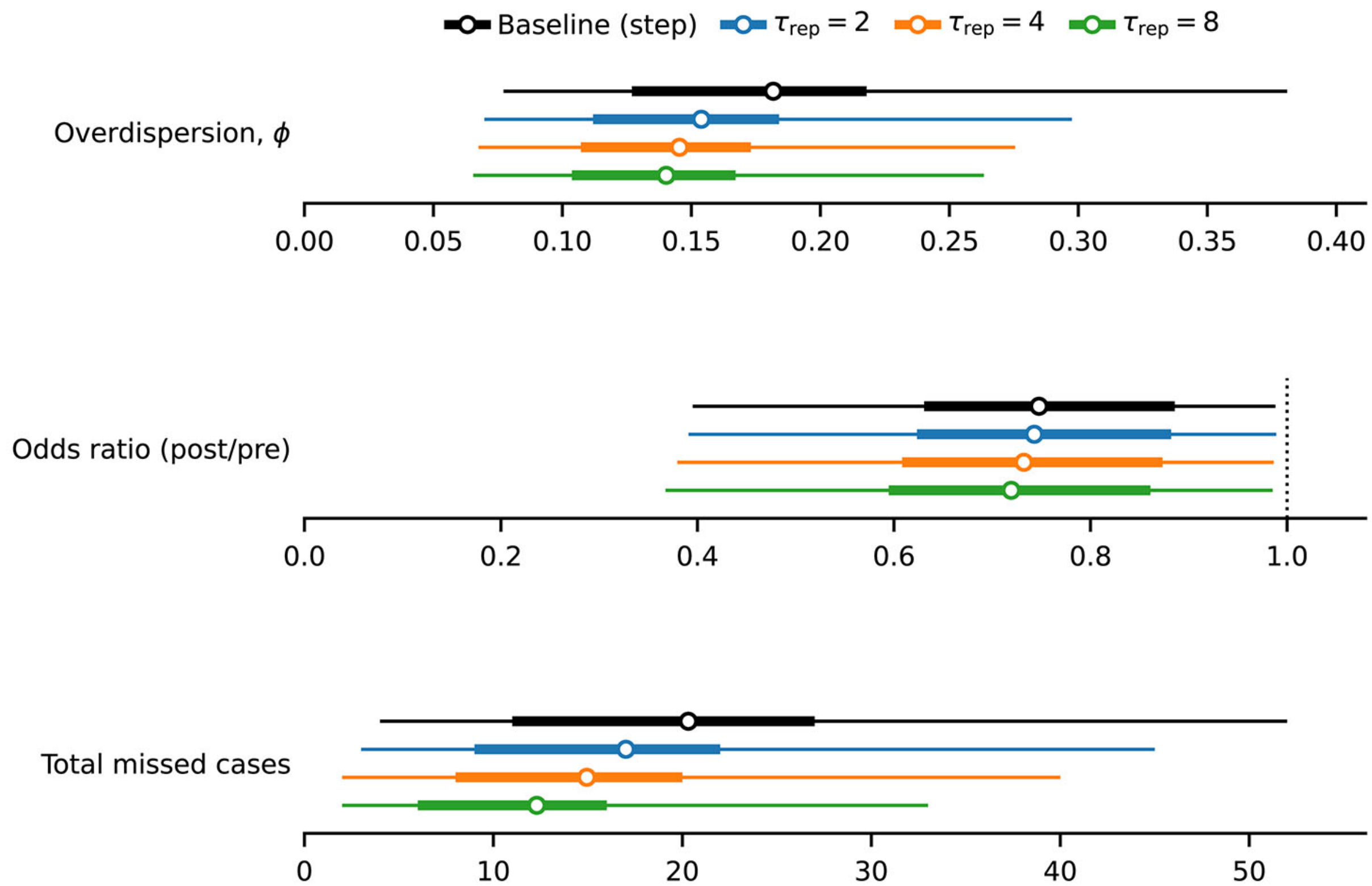

Appendix Figure 3. Sensitivity analysis of complementary model parameters on the time-varying case under-ascertainment parameter, τ_{rep}.

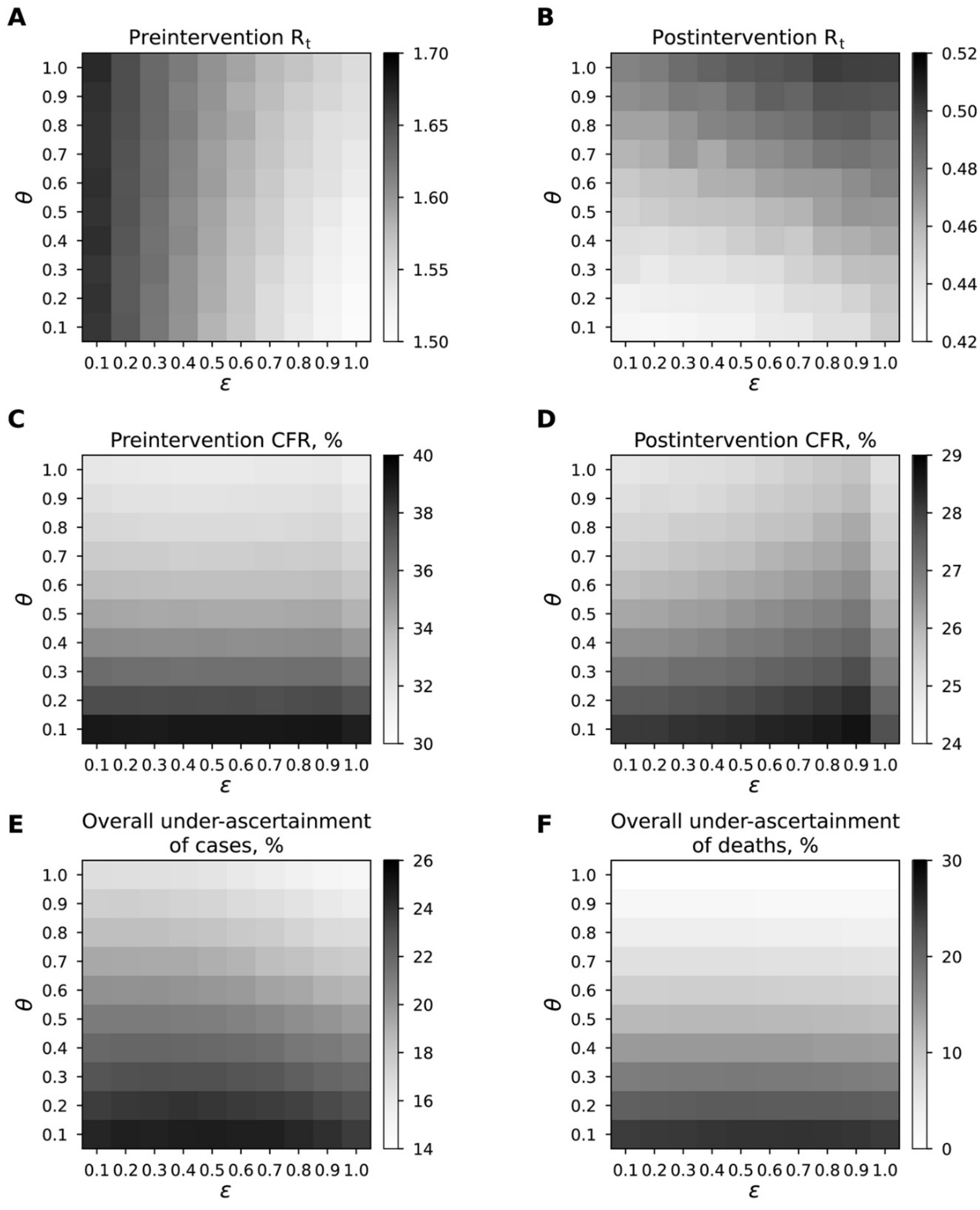

Appendix Figure 4. Sensitivity of parameter estimates to partial ascertainment of both cases and deaths. The baseline model was refitted over a grid of two fixed parameters: ε, the completeness of case ascertainment after week 48, and θ, the completeness of ascertainment of deaths among the unascertained cases. $\varepsilon = \theta = 1$ reproduces the baseline model and is located at the top right cell of each panel. R_{eff}, effective reproduction number; CFR, case-fatality rate.

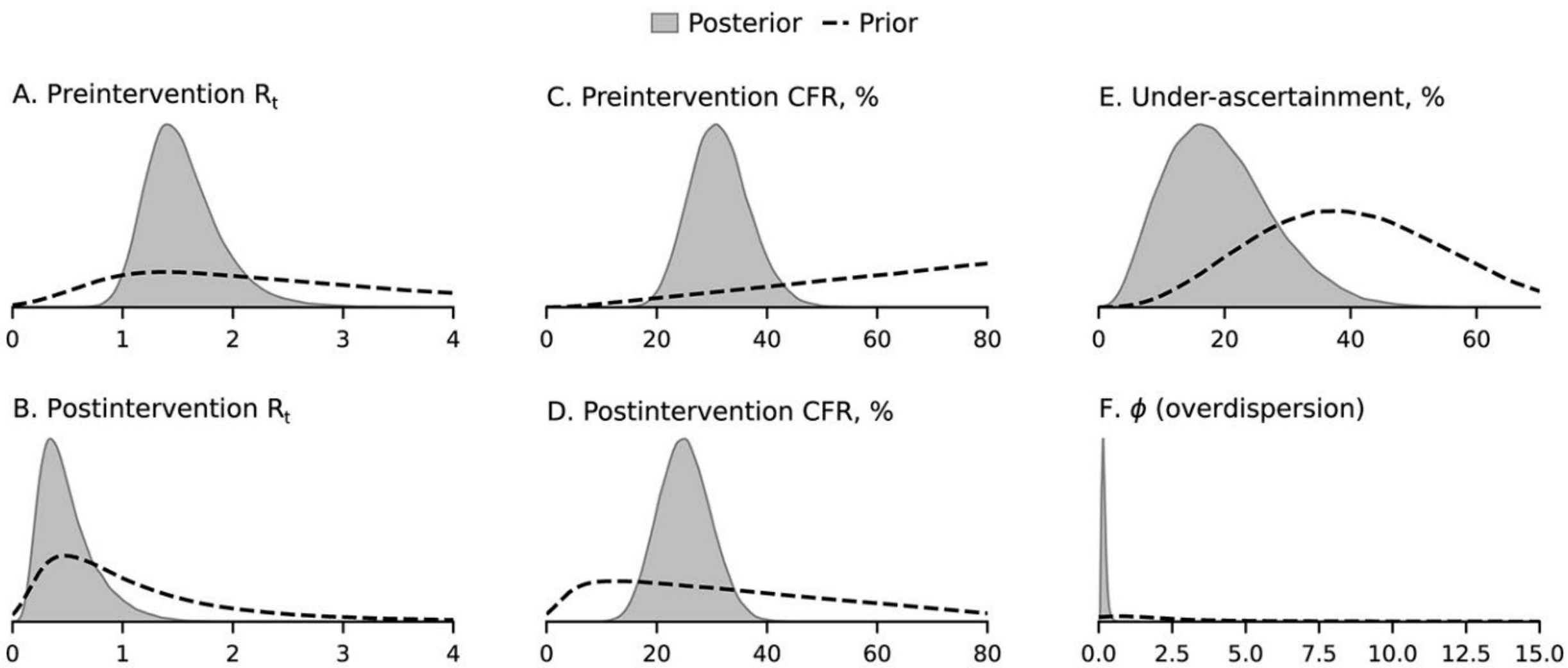

Appendix Figure 5. Comparison of the prior and posterior distributions of the model parameters. R_{eff}, effective reproduction number; CFR, case-fatality rate.

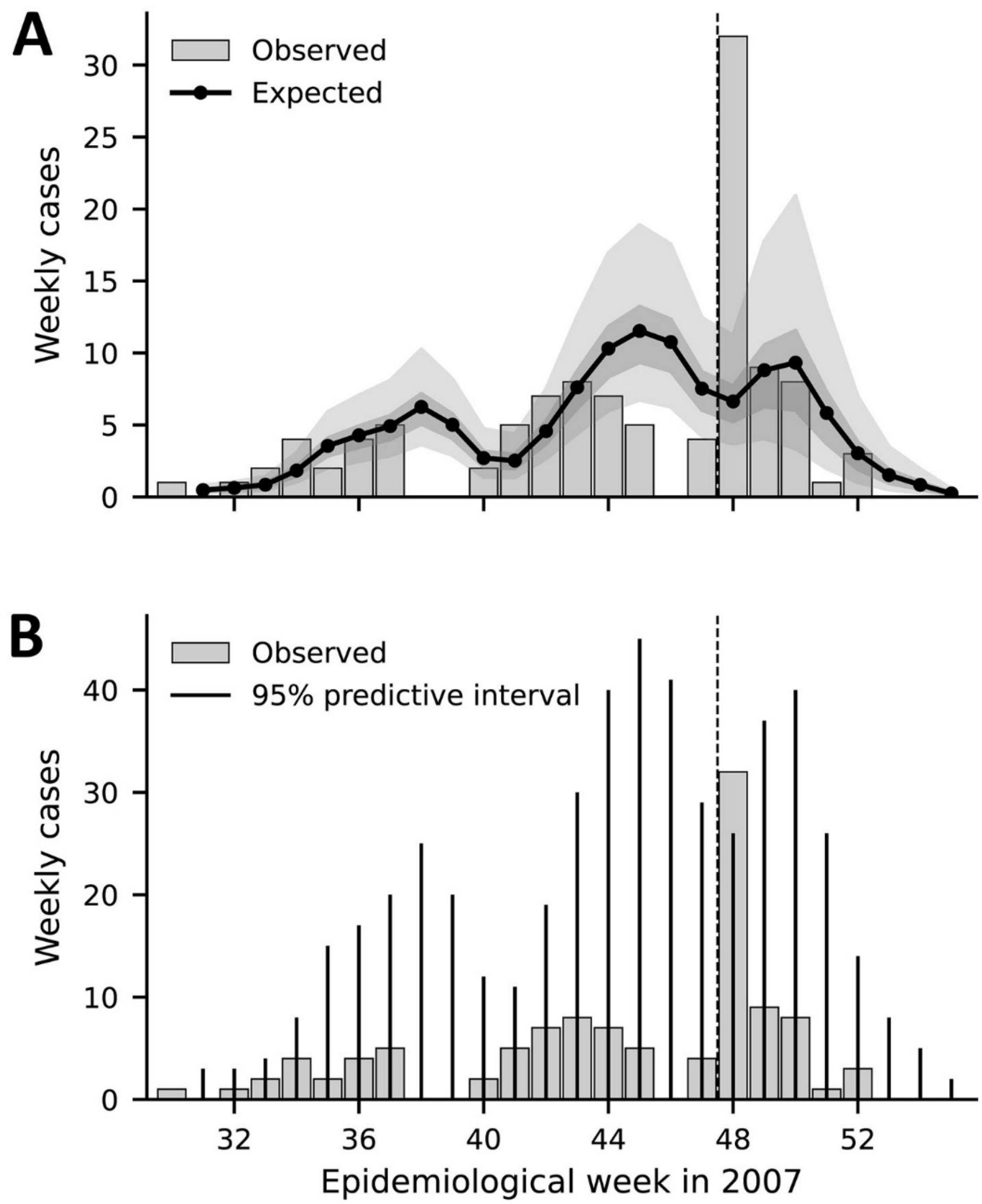

Appendix Figure 6. Model fit (A) and posterior predictive coverage (B) for weekly case counts. In both panels, gray bars show the observed weekly case counts, and the vertical dashed line marks the week control measures were implemented. Solid line in (A) shows the posterior mean and the light and dark shaded areas the 95% and 50% (interquartile) credible intervals.
